# Butterfly eyespot color pattern formation requires physical contact of the pupal wing epithelium with extracellular materials for morphogenic signal propagation

**DOI:** 10.1186/s12861-020-00211-7

**Published:** 2020-03-31

**Authors:** Joji M. Otaki

**Affiliations:** The BCPH Unit of Molecular Physiology, Department of Chemistry, Biology and Marine Science, University of the Ryukyus, Okinawa, 903-0213 Japan

**Keywords:** Butterfly wing, Color pattern formation, Contact angle, Distortion hypothesis, Extracellular matrix, Eyespot, Organizer, Morphogenic signal, Temperature-shock-type modification

## Abstract

**Background:**

Eyespot color pattern formation on butterfly wings is sensitive to physical damage and physical distortion as well as physical contact with materials on the surface of wing epithelial tissue at the pupal stage. Contact-mediated eyespot color pattern changes may imply a developmental role of the extracellular matrix in morphogenic signal propagation. Here, we examined eyespot responses to various contact materials, focusing on the hindwing posterior eyespots of the blue pansy butterfly, *Junonia orithya*.

**Results:**

Contact with various materials, including both nonbiological and biological materials, induced eyespot enlargement, reduction, or no change in eyespot size, and each material was characterized by a unique response profile. For example, silicone glassine paper almost always induced a considerable reduction, while glass plates most frequently induced enlargement, and plastic plates generally produced no change. The biological materials tested here (fibronectin, polylysine, collagen type I, and gelatin) resulted in various responses, but polylysine induced more cases of enlargement, similar to glass plates. The response profile of the materials was not readily predictable from the chemical composition of the materials but was significantly correlated with the water contact angle (water repellency) of the material surface, suggesting that the surface physical chemistry of materials is a determinant of eyespot size. When the proximal side of a prospective eyespot was covered with a size-reducing material (silicone glassine paper) and the distal side and the organizer were covered with a material that rarely induced size reduction (plastic film), the proximal side of the eyespot was reduced in size in comparison with the distal side, suggesting that signal propagation but not organizer activity was inhibited by silicone glassine paper.

**Conclusions:**

These results suggest that physical contact with an appropriate hydrophobic surface is required for morphogenic signals from organizers to propagate normally. The binding of the apical surface of the epithelium with an opposing surface may provide mechanical support for signal propagation. In addition to conventional molecular morphogens, there is a possibility that mechanical distortion of the epithelium that is propagated mechanically serves as a nonmolecular morphogen to induce subsequent molecular changes, in accordance with the distortion hypothesis for butterfly wing color pattern formation.

## Background

Butterfly wing color patterns are very diverse, but they are mostly constructed by placing color pattern elements (or simply elements) in a plain background. The general placement pattern of the elements is known as the nymphalid groundplan [1–3]. Among these elements, eyespots are the most conspicuous and have been targets of developmental studies since the pioneering work of Nijhout (1980) [4]. Because butterfly color patterns are determined during the pupal stage, pupal wing tissues have been studied following physical manipulations using the nymphalid butterfly *Junonia coenia*; cautery (largely equivalent to physical damage) at the center of the putative eyespot results in eyespot size reduction or elimination, and transplantation of the putative eyespot center induces an ectopic eyespot at the transplantation site [4]. These results have since been confirmed in the same and other species in several studies [5–11]. These results have demonstrated that the presumptive eyespot center functions as an organizer inducing eyespot structures to release morphogenic signals that provide specific positional information to immature cells. Organizers are likely located at the center of elements. In addition to the eyespot organizer, the marginal band organizer has been demonstrated by experiments involving physical damage [12]. Interestingly, damage to the background area induces ectopic eyespots [9–11, 13–15].

In addition to these physical manipulations, physiological manipulations can change the location, shape, and size of elements. Elements are modified by temperature shock and chemical application [15–31], and it is likely that similar mechanisms may be employed in the evolution of butterfly wing color patterns [19, 24–31]. Based on the changes in color patterns in response to temperature or chemical application, a possible mechanism of color pattern determination for eyespots and parafocal elements (PFEs; a type of element associated with eyespots) has been proposed as the induction model [32–36]. This model has been applied reasonably well not only to nymphalid butterflies but also to lycaenid butterflies [37].

Furthermore, in addition to these physical and chemical strategies, other approaches have been applied. One such approach is based on classical comparative color pattern analyses. Indeed, the nymphalid groundplan has been determined through color pattern analyses among nymphalid butterflies. This approach may be considered an anatomical approach (including histological and morphometric methods). This line of study has produced several important results in addition to the nymphalid groundplan. This anatomical approach has led to the discovery of butterfly color pattern rules at the elemental and subelemental levels, such as the symmetry rule, the core-paracore rule, the self-similarity rule, the binary rule, the imaginary ring rule, the inside-wide rule, the uncoupling rule, and the midline rule [36]. The examination of scale-size distribution patterns together with color pattern comparisons has revealed high diversity of microscopic (scale-level) color pattern rules that cannot be explained well by a conventional gradient model for positional information, such as the one-cell one-scale rule, the color-size correlation rule, the central maxima rule, the size-ploidy correlation rule, and the distortion rule for organizers [38–40]. The white focal area at the center of an eyespot appears to be independent of the rest of the eyespot [41]. Additionally, the structures of the pupal cuticle spots and the corresponding adult wings have been anatomically studied, revealing the three-dimensionality of pupal and adult wings [8, 42].

Recent technical advancements in two additional approaches (i.e., bioimaging physiology and genome-editing genetics) for studying butterfly wings are notable. Real-time bioimaging techniques for monitoring developing wing epithelial cells have helped to understand their dynamic nature; for example, peripheral adjustment, contraction movements, coloration order, overpainting of colors, elongation of cellular structures, cytoneme-like horizontal processes, and calcium waves have been discovered [43–48]. The CRISPR/Cas9 genome-editing system has led to the functional identification of molecules involved in eyespot development [49–56], which has complemented and fortified previous molecular approaches with analyses of gene expression patterns [57–64], transgenics [64], RNAi [65], and baculovirus-mediated gene transfer [66]. Some of the functionally tested molecules may be considered “molecular morphogens”. However, how these physiological and genetic results are related to physical and chemical experimental results and anatomical findings has remained unknown thus far.

A hypothesis regarding wing color pattern determination that explains a mechanical aspect of wing epithelial is referred to as the distortion hypothesis [36]. The distortion hypothesis suggests that, in addition to the possible molecular morphogens discussed above, the physical distortion of epithelial sheet serves as a “nonmolecular morphogen” that is released directly from an organizer [36]. This hypothesis is based on the following observations. First, eyespot organizers are physically distorted (i.e., the distortion rule for organizers) [8, 42]. On the surface of the pupal dorsal forewing, three-dimensional structures called pupal cuticle spots are constructed just above the organizer, and the underlying epithelia are distorted accordingly. Second, the size of the pupal cuticle spots is roughly proportional to adult eyespot size [42]. Third, eyespot color patterns are sensitive not only to physical damage but also to physical distortion of the wing epithelium [11, 67].

Although the distortion hypothesis should be examined experimentally, another interesting aspect of eyespot changes is that eyespots respond to contact with materials on the wing tissue at the pupal stage [67]. Focusing on the hindwing eyespots of the peacock pansy butterfly, *Junonia almana*, it has been demonstrated that its minor eyespots respond differently to contact with materials such as plastic film, glass, silicone glassine paper, and medical adhesive tape; the latter two materials exhibit a strong eyespot-reducing ability. Interestingly, parafocal elements shift toward the nearest eyespot center, which is an important characteristic of color pattern modifications induced by temperature shock and chemical application (i.e., TS-type modifications) [15–31]. These results suggest that contact with the surface of epithelial cells plays an important role in the propagation of nonmolecular morphogenic signals or in organizer activity itself. However, the previous experimental data concerning contact-mediated eyespot modifications are not sufficient to fully understand the response pattern of eyespots systematically.

**Fig. 1:**
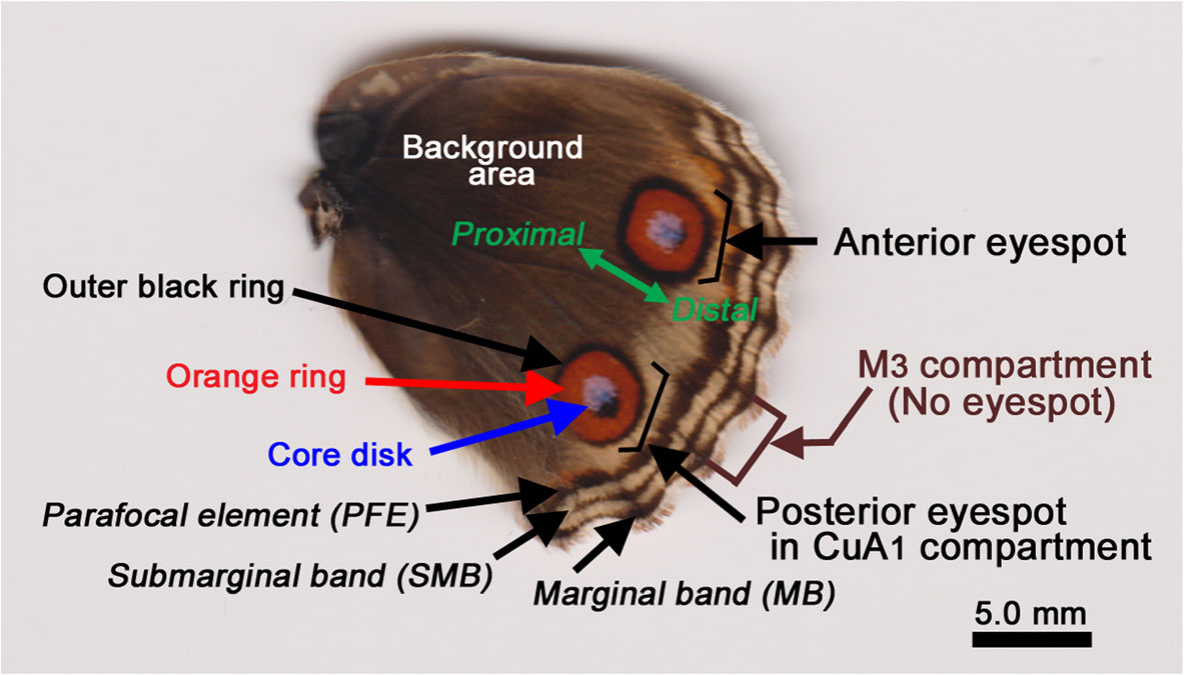
Nomenclature of the dorsal hindwing of *Junonia orithya*. The posterior eyespot (located in the CuA_1_ compartment) is the target of this study, and the anterior eyespot is not. An eyespot is composed of a core disk, orange ring, and outer black ring. In addition to eyespots, parafocal elements (PFEs) and, less frequently, submarginal bands (SMBs) are other elements that are affected by the contact treatments. The M_3_ compartment does not have an eyespot

In the present study, I introduced new materials and methods. First, I focused on a different but related butterfly species, *Junonia orithya* (Fig. 1). Because this species has a large posterior eyespot on its hindwing, which is the target of the present study, eyespot responses were more robust than those of *J. almana* used in the previous study [67]. Second, various materials (17 treatment modes in contrast to just 5 in the previous study [67]) were tested. Third, physicochemical properties of various materials were measured according to the water contact angle of the material surface, which represents the repellency or hydrophobicity of the material surface. Fourth, I statistically evaluated relationships between eyespot change and contact angle. Fifth, I then demonstrated that the material surface contact was required for morphogenic signal propagation, using two contact materials simultaneously. The possible contributions of the materials’ surface properties to eyespot signal propagation are discussed in light of the distortion hypothesis.

## Results

### Epithelial contact was required for eyespot development

**Fig. 2:**
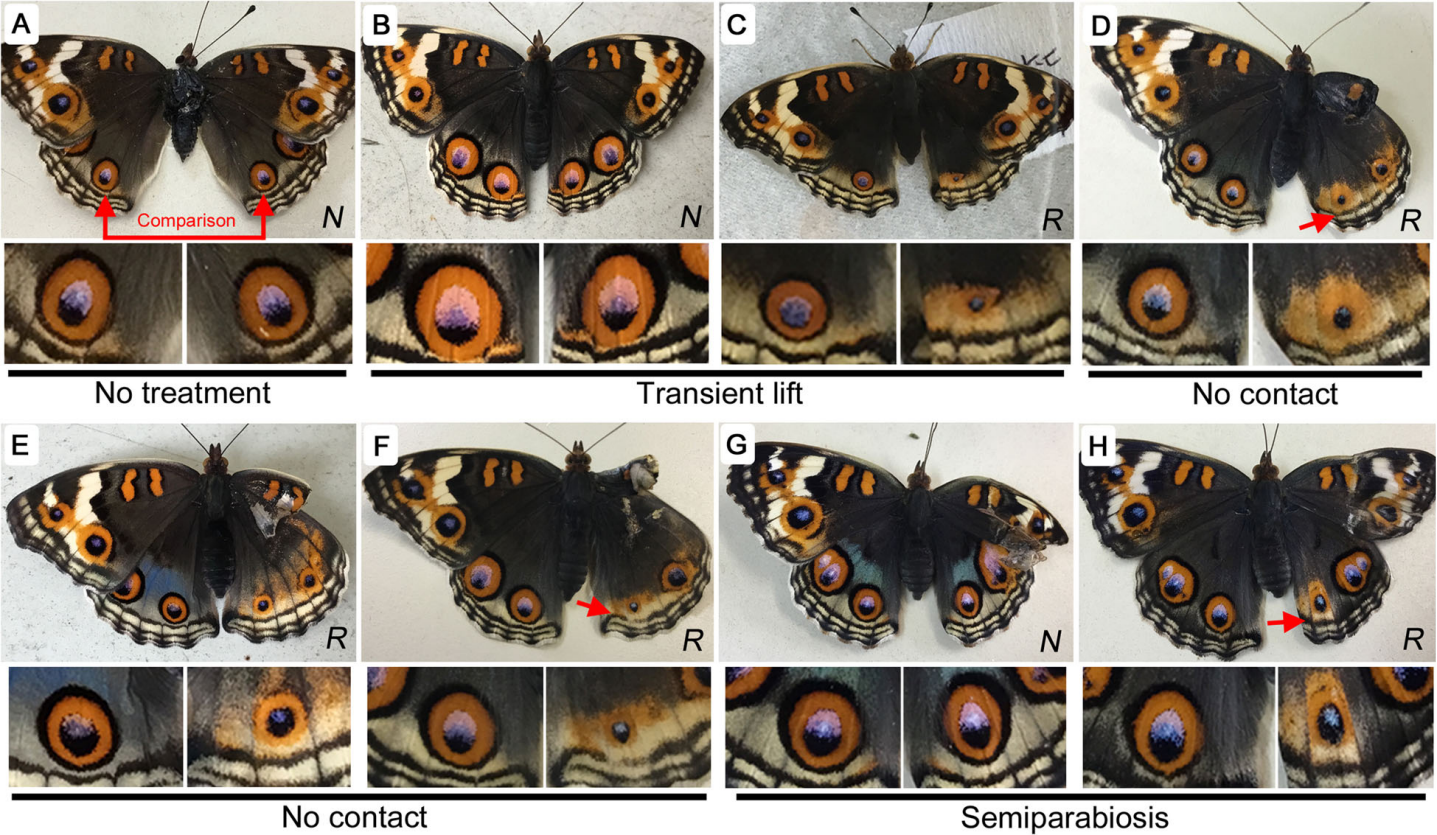
Wing-lift-induced changes in eyespot size. *N* (no change) or *R* (reduction) is indicated at the bottom right in each panel. Red arrows indicate modified parafocal elements (PFEs). Right (experimental) and left (control) posterior eyespots are enlarged below a whole butterfly image. **a** No treatment. The right and left posterior eyespots are compared in size throughout this study. **b**, **c** Transient lift treatment. **d-f** No contact treatment. **g**, **h** Semiparabiosis

**Fig. 3:**
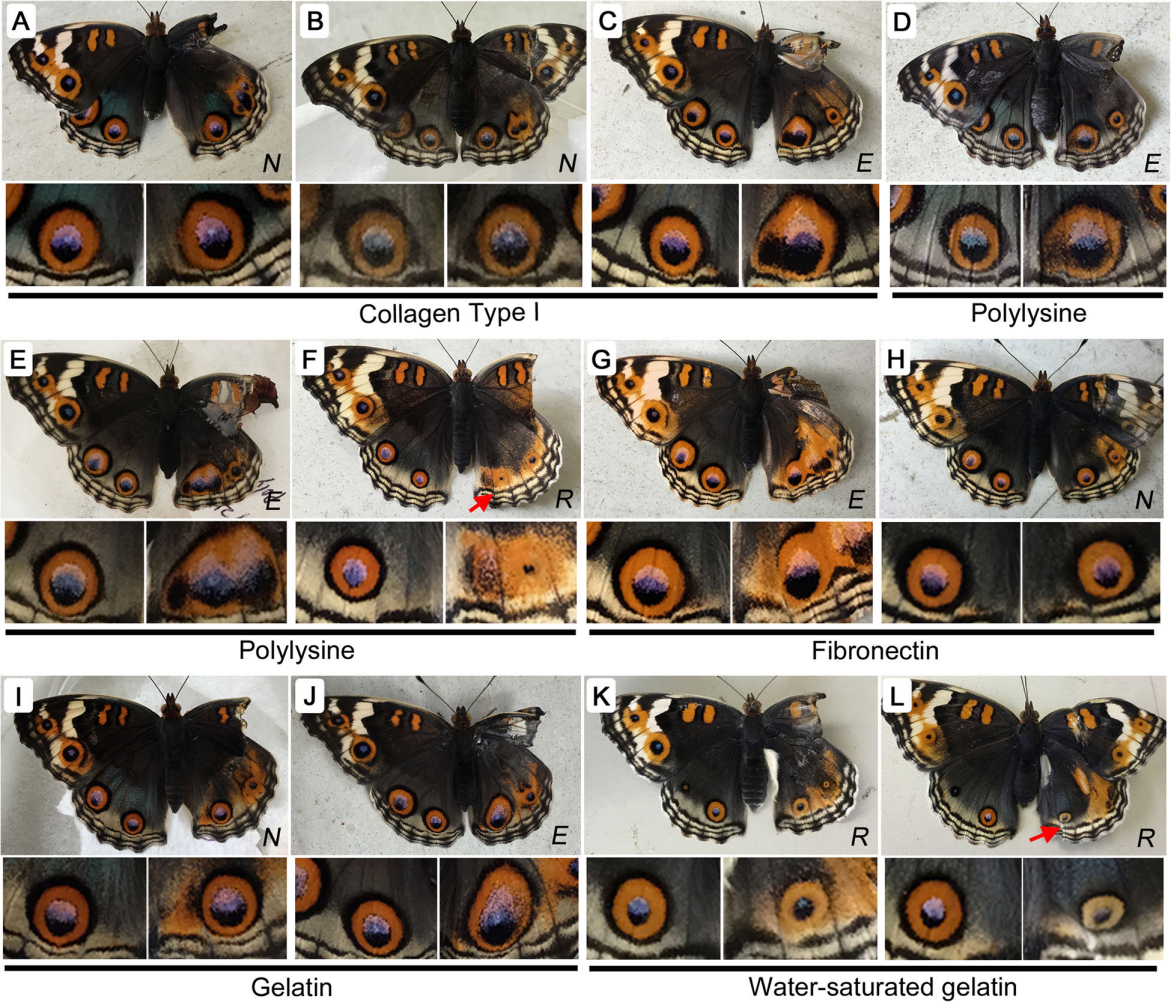
Changes in eyespot size induced by biological contact materials. *N* (no change), *E* (enlargement), or *R* (reduction) is indicated at the bottom right in each panel. Red arrows indicate modified parafocal elements (PFEs). Right (experimental) and left (control) posterior eyespots are enlarged below a whole butterfly image. **a-c** Collagen Type I. **d-f** Polylysine. **g**, **h** Fibronectin. **i**, **j** Gelatin. **k**, **l** Water-saturated gelatin

In butterflies, the color patterns on the right and left wings are generally symmetric in an individual. Not surprisingly, when the right posterior eyespot was compared with the left eyespot in the no treatment control group (*n* = 19), they were found to be identical in size in all individuals examined (Fig. 2a). When the forewing was lifted transiently and then returned to the original position in approximately 1 minute (*n* = 42), no effect was observed in the majority of individuals (*n* = 25) (Fig. 2b). However, in some individuals, the eyespot was enlarged in size (*n* = 15) (not shown for the transient lift treatment, but see similar cases of eyespot enlargement in Fig. 3c, d as examples). Additionally, in rare cases, the eyespot was extensively reduced in size (*n* = 2) (Fig. 2c). This response variability under the transient lift treatment might have arisen from variable degrees of physical contact of the dorsal hindwing eyespot with the repositioned ventral forewing, likely because of small wrinkles in the forewing unintentionally introduced by the manipulation.

The extensive reduction observed in rare cases above was “reproduced” when the hindwing epithelium experienced no physical contact with any material; all individuals under this no contact treatment consistently showed extensively reduced eyespots (*n* = 20) (Fig. 2d-f). Furthermore, the “semiparabiosis” treatment, in which the forewing from a different individual was used as the contact material, produced results similar to the transient lift treatment (*n* = 16); the majority of individuals showed no change (*n* = 10), but the eyespots of a minority of individuals were either enlarged (*n* = 4) or reduced (*n* = 2) in size (Fig. 2g, h). This level of variability may be unavoidable for most such materials, probably because of the variability of the physical contact with the epithelium.

Interestingly, highly reduced eyespots were often associated with thickening (or blurring) of parafocal elements (PFEs) dislocated toward the proximal side (Fig. 2d, f, h). This dislocation of PFEs is a prominent characteristic that has been observed in modifications induced by temperature shock [15], tungstate [16], or heparin and other related chemicals [23]. On the other hand, eyespot location within the CuA_1_ compartment appeared to be invariable. Eyespot shape did not seem to be affected much, either. However, in miniaturized eyespots, the proportions of the eyespot subelements (i.e., outer black ring, orange ring, and core disk) appeared to differ from those of nontreated ones. The outer black ring disappeared more often than others, and the orange ring often diffused. These changes of PFE and eyespot in location and shape observed here were also observed in subsequent treatments.

### Biological materials induced various eyespot responses

Here, biological materials employed as plate-coating reagents were used as contact materials. Collagen type I (*n* = 27) induced eyespot enlargement in many individuals (*n* = 11), although the degree of enlargement was not high (Fig. 3a-c). No change (*n* = 6) or a reduction (*n* = 10) was observed in some individuals subjected to collagen treatment as well. Polylysine (*n* = 21) also produced various enlargements (*n* = 11) or reductions (*n* = 7) or no change (*n* = 3) in some individuals (Fig. 3d-f). Fibronectin (*n* = 26) produced no change in the greatest number of individuals (*n* = 14), followed by reductions (*n* = 6) and enlargements (*n* = 6) (Fig. 3g, h). Gelatin (*n* = 46) produced reductions (*n* = 19) or no change (*n* = 18) at almost equal frequencies (Fig. 3i, j) and a lower frequency of enlargements (*n* = 9). Overall, these 4 biological materials resulted in enlargements, reductions, and no change in different ratios. When gelatin coating was saturated with water (*n* = 31), most individuals showed reduced eyespots (*n* = 19) or no change (*n* = 11), and only one individual showed enlargement (*n* = 1) (Fig. 3k, l), probably because firm contact with the epithelium was blocked due to the thin water layer covering the gelatin layer. Overall, enlarged eyespots were elongated toward the proximal direction, often deformed, and fused with adjacent ectopic eyespots, which were also observed in subsequent treatments.

### Nonbiological materials induced various eyespot responses

**Fig. 4:**
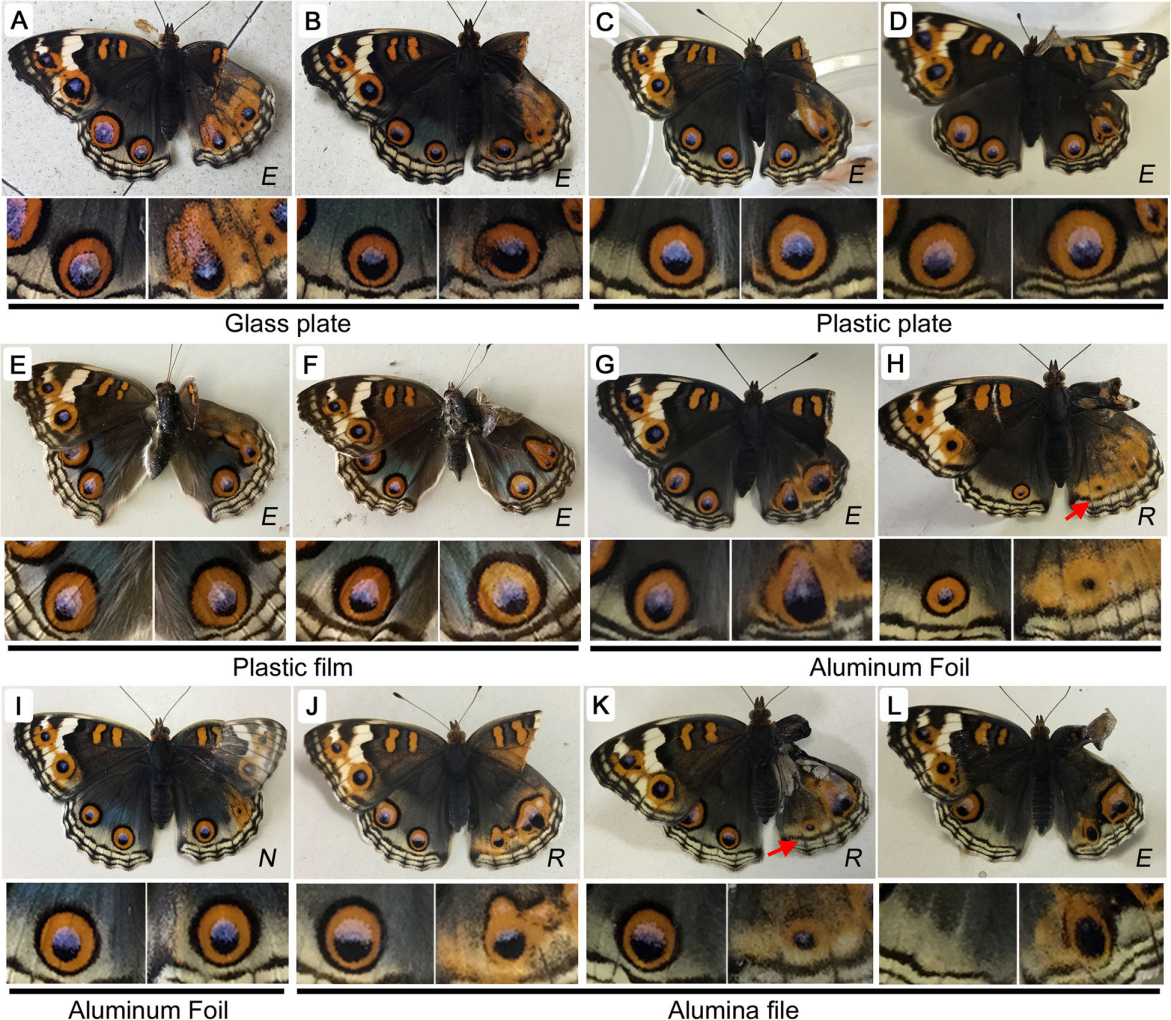
Changes in eyespot size induced by various nonbiological contact materials. *N* (no change), *E* (enlargement), or *R* (reduction) is indicated at the bottom right in each panel. Red arrows indicate modified parafocal elements (PFEs). Right (experimental) and left (control) posterior eyespots are enlarged below a whole butterfly image. **a**, **b** Glass plate. **c**, **d** Plastic plate. **e**, **f** Plastic film. **g-i** Aluminum foil. **j-l** Alumina file

Next, nonbiological materials were tested as contact materials. Glass plates (*n* = 21) appeared to increase eyespot size in more individuals (*n* = 12) than any other material tested (Fig. 4a, b), although they also produced no change (*n* = 7) or reductions (*n* = 2). In contrast, plastic plates (*n* = 23) mostly produced no change (*n* = 18), followed by enlargements (*n* = 4) and a reduction (*n* = 1) (Fig. 4c, d), and this material resulted in no change at the highest frequency other than the absence of treatment. Plastic film (*n* = 38) showed similar results to plastic plates, leading to no change (*n* = 25), enlargements (*n* = 12), or a reduction (*n* = 1) (Fig. 4e, f). The fact that plastic plates and plastic film resulted in similar profiles suggests that the physical rigidity of the contact materials does not strongly affect eyespot development. In contrast, when aluminum foil (*n* = 53) was tested, the majority of individuals showed no change (*n* = 27), with only small proportions of enlargements (*n* = 16) and reductions (*n* = 10) (Fig. 4g-i).

For comparison, alumina file (composed of alumina powder; see Materials and Methods) was tested as a contact material (*n* = 46). All 3 categories of results were again obtained, similar to the results obtained with aluminum foil: enlargements (*n* = 10), reductions (*n* = 29), or no change (*n* = 7) (Fig. 4j-l). However, the ratios resulting from alumina file treatment were very different from those for aluminum foil.

An unexpected finding was that the alumina file treatment induced a posterior eyespot in a treated individual that lacked the posterior eyespot likely due to a genetic mutation (*n* = 1) (Fig. 4l). This no-eyespot mutant emerged incidentally, and this result was unexpected but importantly suggests the contribution of the extracellular matrix (ECM) to eyespot size in natural populations of *J. orithya*.

### Some nonbiological materials induced considerable eyespot size reduction

**Fig. 5:**
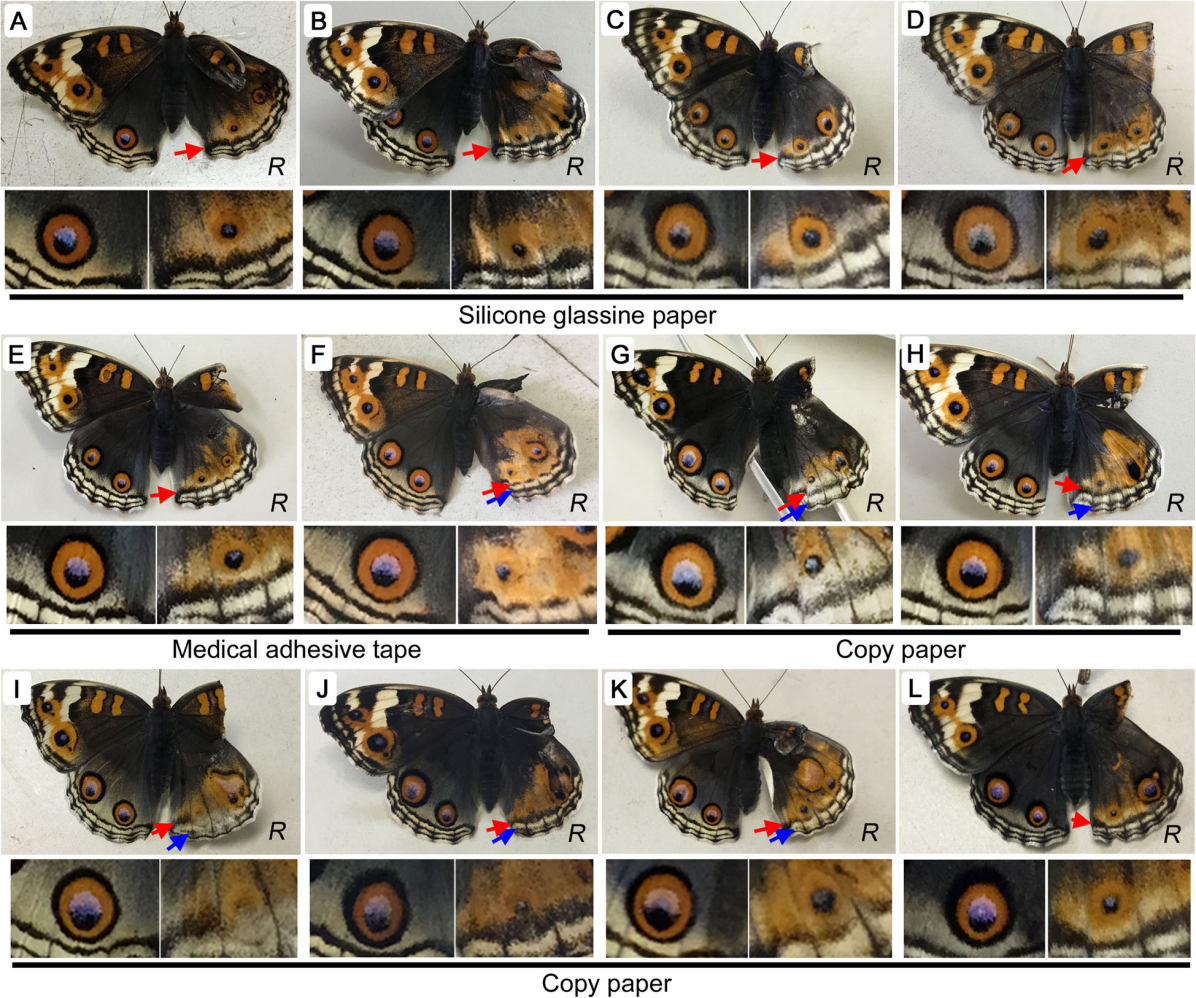
Changes in eyespot size induced by additional nonbiological contact materials. *R* (reduction) is indicated at the bottom right in each panel. Red arrows indicate modified parafocal elements (PFEs), and blue arrows indicate modified submarginal bands (SMBs). Right (experimental) and left (control) posterior eyespots are enlarged below a whole butterfly image. **a-d** Silicone glassine paper. **e**, **f** Medical adhesive tape. **g-l** Copy paper

To further examine the possible effects of the contact material surface, additional nonbiological materials were tested. First, silicone glassine paper, which has a smooth slippery surface, was tested (*n* = 26) and resulted in highly reduced eyespots in almost all individuals (*n* = 25), with a single exception showing no change (*n* = 1) (Fig. 5a-d). Second, medical adhesive tape was tested (*n* = 20), which is very sticky on dry surfaces but not at all sticky on wet surfaces, again resulting in highly reduced eyespots in all individuals (Fig. 5e, f). Third, when copy paper was tested (*n* = 42), very small eyespots were obtained in all treated individuals (Fig. 5g-l). In all 3 cases, PFEs were shifted toward the proximal side, which is a prominent characteristic of TS-type (temperature-shock-type) modifications. Copy paper treatment appeared to cause the most intensive dislocation and thickening of PFEs. Associated with the PFE dislocation, submarginal bands (SMBs) were blurred and thickened in some individuals.

### Evaluation of the effects of treatments

**Fig. 6:**
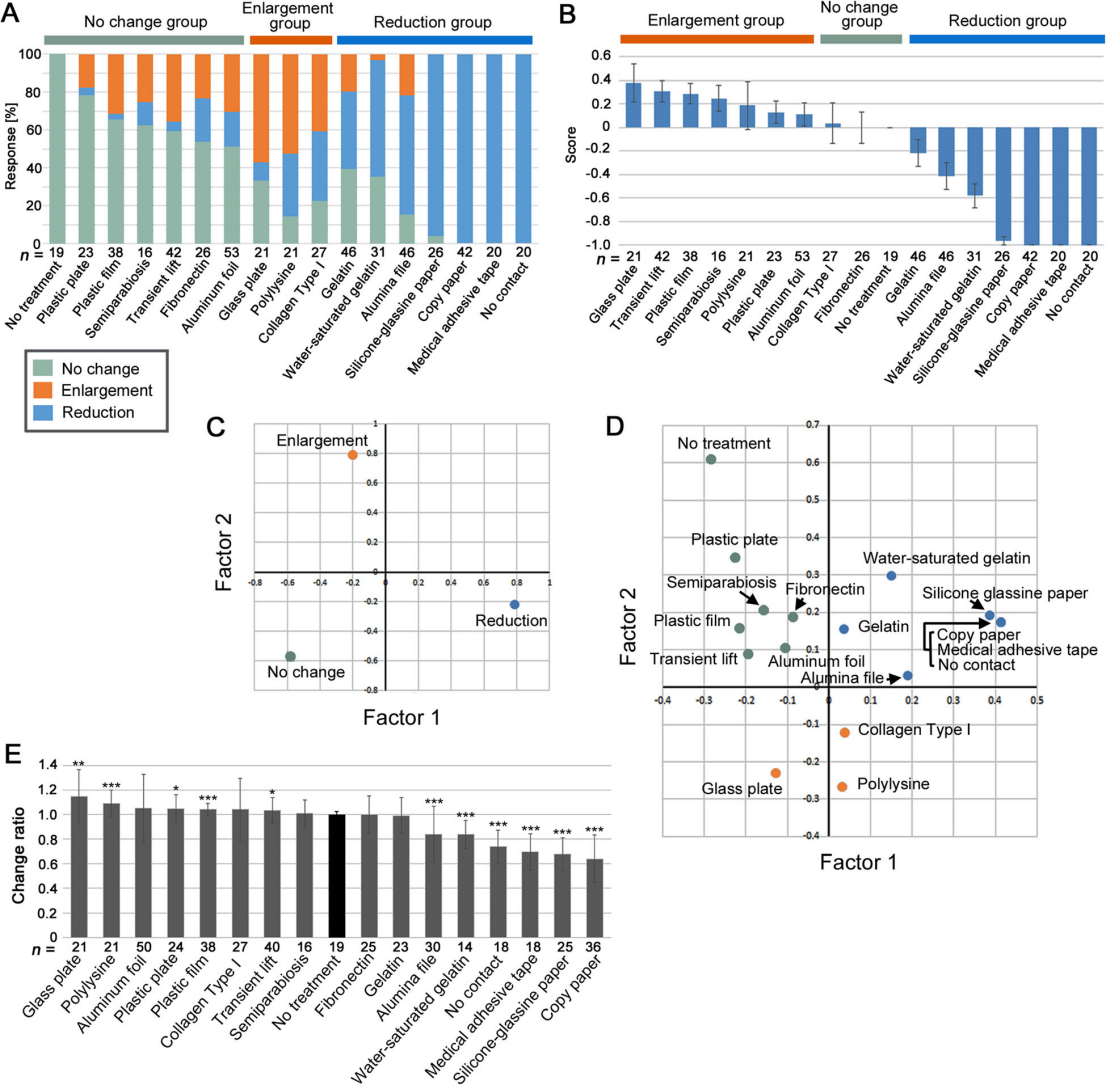
Quantitative analyses of eyespot changes. **a** Response profiles of 17 treatment modes. Response results (no change, enlargement, and reduction) vary among treatment modes. Based on the result that was obtained most frequently, the treatments could be classified into 3 groups: no change group, enlargement group, and reduction group. **b** Eyespot change scores of 17 treatment modes. No change, enlargement, and reduction received scores of 0, 1, and − 1, respectively. The mean and standard error are shown. Treatments with positive mean scores are considered to belong to the enlargement group. Treatments with negative mean scores are considered to belong to the reduction group. Treatments with zero or near-zero mean scores are considered to belong to the no change group. **c** PCA plot of the 3 treatment modes in two-dimensional space using Factor 1 (eigenvalue 2279; variance ratio = 0.846) and Factor 2 (eigenvalue = 414; variance ratio = 0.154). They are well separated. **d** PCA plot of 17 treatment modes in two-dimensional space using Factor 1 (eigenvalue 18,659; variance ratio = 0.792) and Factor 2 (eigenvalue = 4891; variance ratio = 0.208). The groups defined by the profiles in A are well reflected. **e** Eyespot size change ratio of 17 treatment modes. Each treatment mode was compared with no treatment. ***: *p* < 0.001, **: *p* < 0.01, *: *p* < 0.05 (based on raw *p*-values without Bonferroni or other correction)

To compare the effects of the contact materials tested in this study, the percentages of enlargement, reduction, and no change under each treatment, including the control treatments (referred to as the response profile), were compiled (Fig. 6a; Tables S1, S2). Reduction in all cases (100%) or nearly all cases was observed under treatment with 3 materials (copy paper, medical adhesive tape, and silicone glassine paper) and the no contact treatment. In contrast, enlargement was never achieved in all cases under any treatment. The highest percentage of enlargement was observed for glass plates (57.1%) and polylysine (52.4%), followed by collagen type I (40.7%), a transient lift (35.7%), and plastic film (17.4%). No change was achieved in most individuals under treatment with plastic plates (78.3%) and plastic film (65.8%). For convenience, the treatment modes were grouped into 3 categories (no change group, enlargement group, and reduction group) based on the most frequent response mode under a treatment (Fig. 6a).

Each treatment was evaluated quantitatively by assigning change scores (Fig. 6b; Tables S1, S2). Biological materials such as gelatin (mean ± standard error = − 0.22 ± 0.11), fibronectin (0.00 ± 0.14) and collagen type I (0.04 ± 0.17) showed near-zero values. Polylysine (0.19 ± 0.20), on the other hand, showed a more positive (enlargement) value, and gelatin water (− 0.58 ± 0.10) showed a more negative (reduction) value. Aluminum foil (0.11 ± 0.10) and alumina file (− 0.41 ± 0.12) showed positive and negative values, respectively, despite their relatively similar chemical compositions. Again, for convenience, the treatment modes were grouped into 3 categories (no change group, enlargement group, and reduction group) based on showing positive, near-zero, and negative scores, respectively (Fig. 6b), which was somewhat different from the previous categorization (Fig. 6a).

The relationships among the 3 response types (i.e., no change, enlargement, and reduction) were examined by principal component analysis (PCA) (Fig. 6c). The 3 responses were well separated from one another in two-dimensional space. The relationships among the treatment modes were also examined by PCA (Fig. 6d). The treatment modes that belonged to the same group defined in Fig. 6a were plotted in the same area in two-dimensional space, despite that a given group contained chemically different materials.

Quantitative evaluation of eyespot change ratio (the proximodistal size on the midline of the treated right eyespot divided by that of the corresponding nontreated left eyespot) (Table S3) indicated that copy paper (mean ± standard deviation = 0.641 ± 0.194; *t* = 10.9, *df* = 37, *p* < 0.0001), silicone glassine paper (0.678 ± 0.132; *t* = 11.9, *df* = 26, *p* < 0.0001), medical adhesive tape (0.696 ± 0.146; *t* = 8.7, *df* = 18, *p* < 0.0001), no contact (0.739 ± 0.135; *t* = 8.1, *df* = 18, *p* < 0.0001), water-saturated gelatin (0.837 ± 0.118; *t* = 5.0, *df* = 14, *p* = 0.0002), and alumina file (0.841 ± 0.228; *t* = 3.8, *df* = 30, *p* = 0.0007) produced significantly smaller eyespot than no treatment (0.999 ± 0.025) (Fig. 6e). In contrast, glass plates (1.148 ± 0.218; *t* = 3.1, *df* = 21, *p* = 0.0053), polylysine (1.093 ± 0.106; *t* = 4.0, *df* = 22, *p* = 0.0007), plastic plates (1.050 ± 0.114; *t* = 2.1, *df* = 26, *p* = 0.044), plastic film (1.042 ± 0.050; *t* = 4.3, *df* = 55, *p* < 0.0001), and transient lift (1.034 ± 0.103; *t* = 2.0, *df* = 48, *p* = 0.046) produced significantly larger eyespot than no treatment (Fig. 6e).

A pair of gelatin (0.993 ± 0.144) and water-saturated gelatin showed a significant difference in eyespot size (*t* = 3.4, *df* = 35, *p* = 0.0017), suggesting that water-mediated inhibition of direct binding of epithelial cells to gelatin caused eyespot size reduction. A pair of aluminum foil (1.054 ± 0.275) and alumina file (0.841 ± 0.228) also showed significant difference (*t* = 3.6, *df* = 78, *p* = 0.0006), suggesting importance of surface structures (but not chemical compositions). In contrast, a pair of plastic plate (1.050 ± 0.114) and plastic film (1.042 ± 0.050) did not show significant difference (*t* = 0.32, *df* = 29, *p* = 0.75), suggesting unimportance of material rigidity.

### Water contact angles of the contact materials

**Fig. 7:**
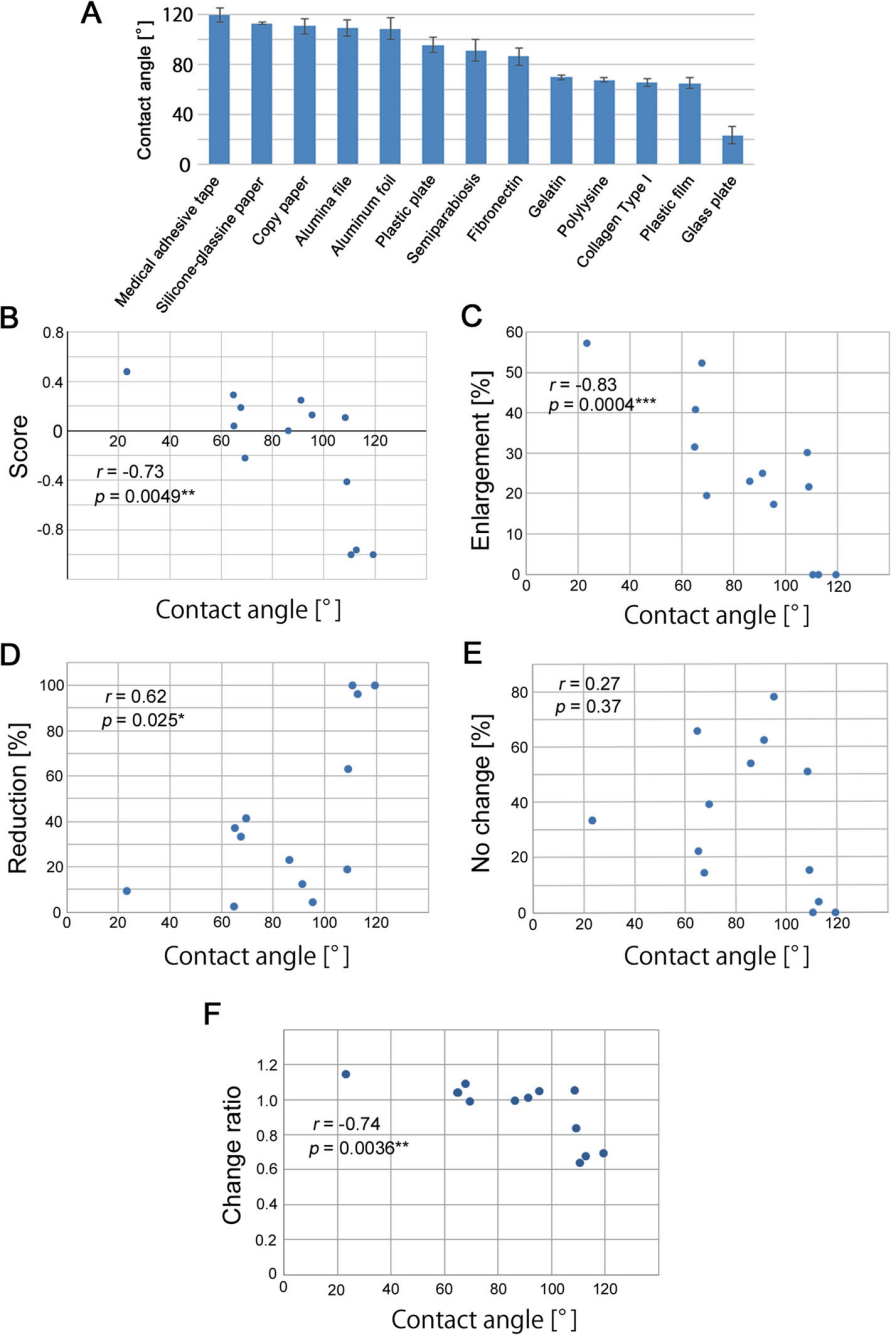
Contact angles and their relationship with eyespot responses. **a** Contact angles of 13 materials used in this study. **b** Scatter plot and correlation coefficient between the contact angle and eyespot response score. **c** Scatter plot and correlation coefficient between the contact angle and enlargement percent. **d** Scatter plot and correlation coefficient between the contact angle and reduction percent. **e** Scatter plot and correlation coefficient between the contact angle and no change percent. **f** Scatter plot and correlation coefficient between the contact angle and eyespot size change ratio. Asterisks indicate statistical significance

It appeared that the physicochemical properties of the contact materials, but not their chemical compositions, likely played a role in mediating eyespot changes. The water contact angles of the contact materials were measured, including the dorsal side of the pupal hindwing tissue immediately after pupation (Fig. 7a; Table S2). The contact angles varied from the least water repellant material of glass plates (23.2 ± 6.6°) to the most water repellent material of medical adhesive tape (119.3 ± 5.7°).

Correlation analyses between the contact angle and eyespot size effects were performed. The eyespot change score showed a negative correlation with the contact angle (*r* = − 0.73; *p* = 0.0049) (Fig. 7b). The enlargement percentage also showed a negative correlation (*r* = − 0.83; *p* = 0.0004) (Fig. 7c), indicating that the more water repellant a material was, the smaller the resultant eyespot size. Consistent with this result, the reduction percentage showed a positive correlation (*r* = 0.62; *p* = 0.025) (Fig. 7d). The no change percentage showed no correlation (*r* = 0.27; *p* = 0.37) (Fig. 7e). A negative correlation between contact angle and eyespot change ratio was significant (*r* = − 0.74, *p* = 0.0036) (Fig. 7f). It appeared that the physicochemical properties of contact materials, represented by the water contact angle, were an important determinant of eyespot size in the contact experiments.

### Ectopic eyespot was induced in the M_3_ compartment

**Fig. 8:**
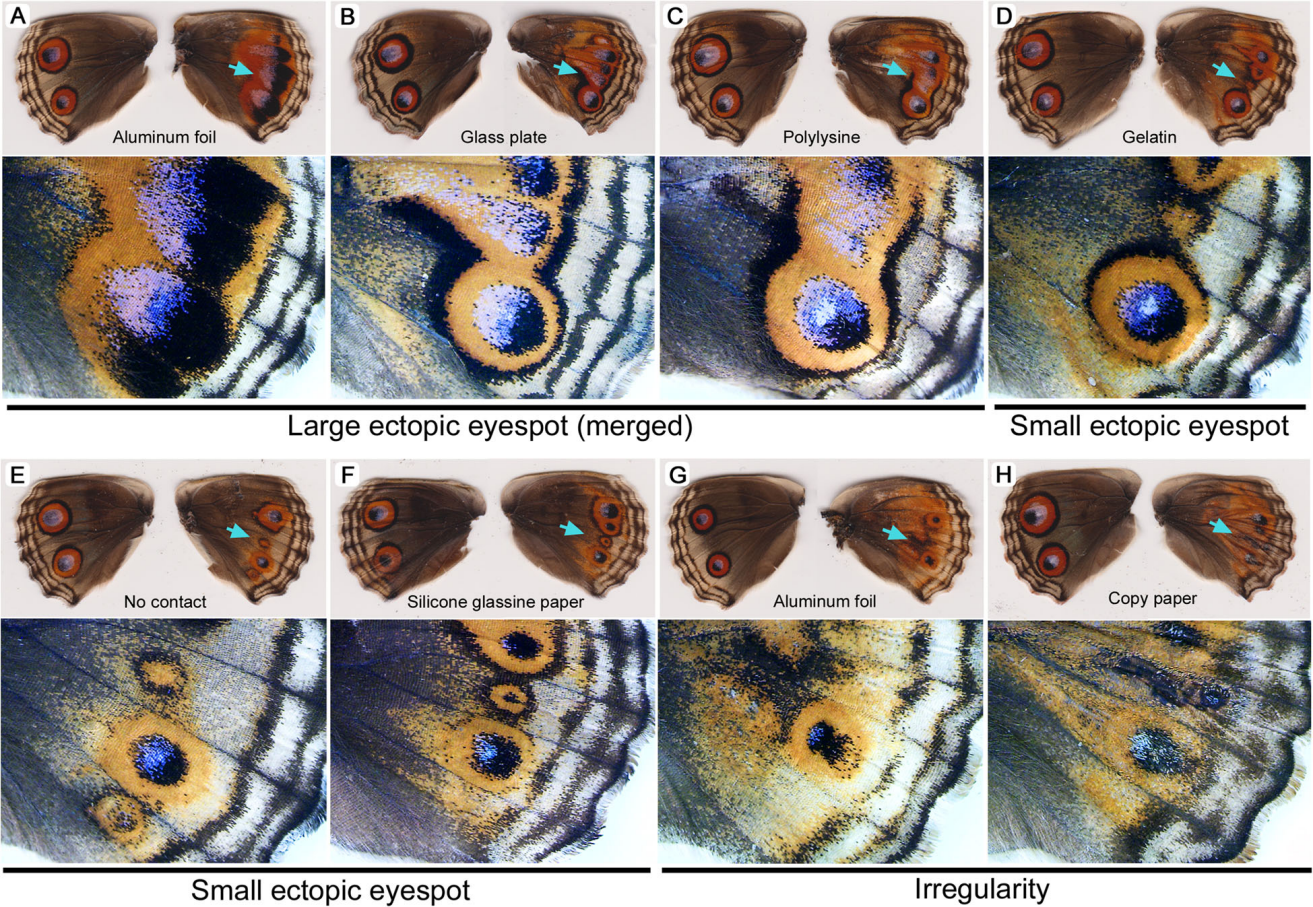
Ectopically induced eyespot in the M_3_ compartment. Both right (experimental) and left (control) wings are shown. Light blue arrows indicate the M_3_ compartment, which is enlarged below the image of the entire wings. Changes in the posterior eyespot in the CuA_1_ compartment are also notable. **a-c** Large ectopic eyespot that merged with the adjacent eyespots. **d-f** Small ectopic eyespot. **g**, **h** Irregular modifications

It is interesting to examine how contact materials influence the M_3_ compartment, where no eyespot exists. Although not quantified, in response to contact materials such as aluminum foil, glass plates, and polylysine, a large ectopic eyespot occasionally emerged, in which case it was often elongated and merged with adjacent eyespots (Fig. 8a-c). In response to contact materials such as gelatin, no contact, and silicone glassine paper, a small ectopic eyespot emerged (Fig. 8d-f). The focus of ectopic eyespot in the M_3_ compartment was often located at the similar position of the CuA_1_ eyespot, but in one case, the focus of ectopic eyespot appeared to be dislocated toward the proximal side (Fig. 8e). Irregular black and orange scales were found in some individuals in response to contact materials such as aluminum foil and copy paper (Fig. 8g, h).

### Uneven contact experiments revealed that signal propagation was inhibited

**Fig. 9:**
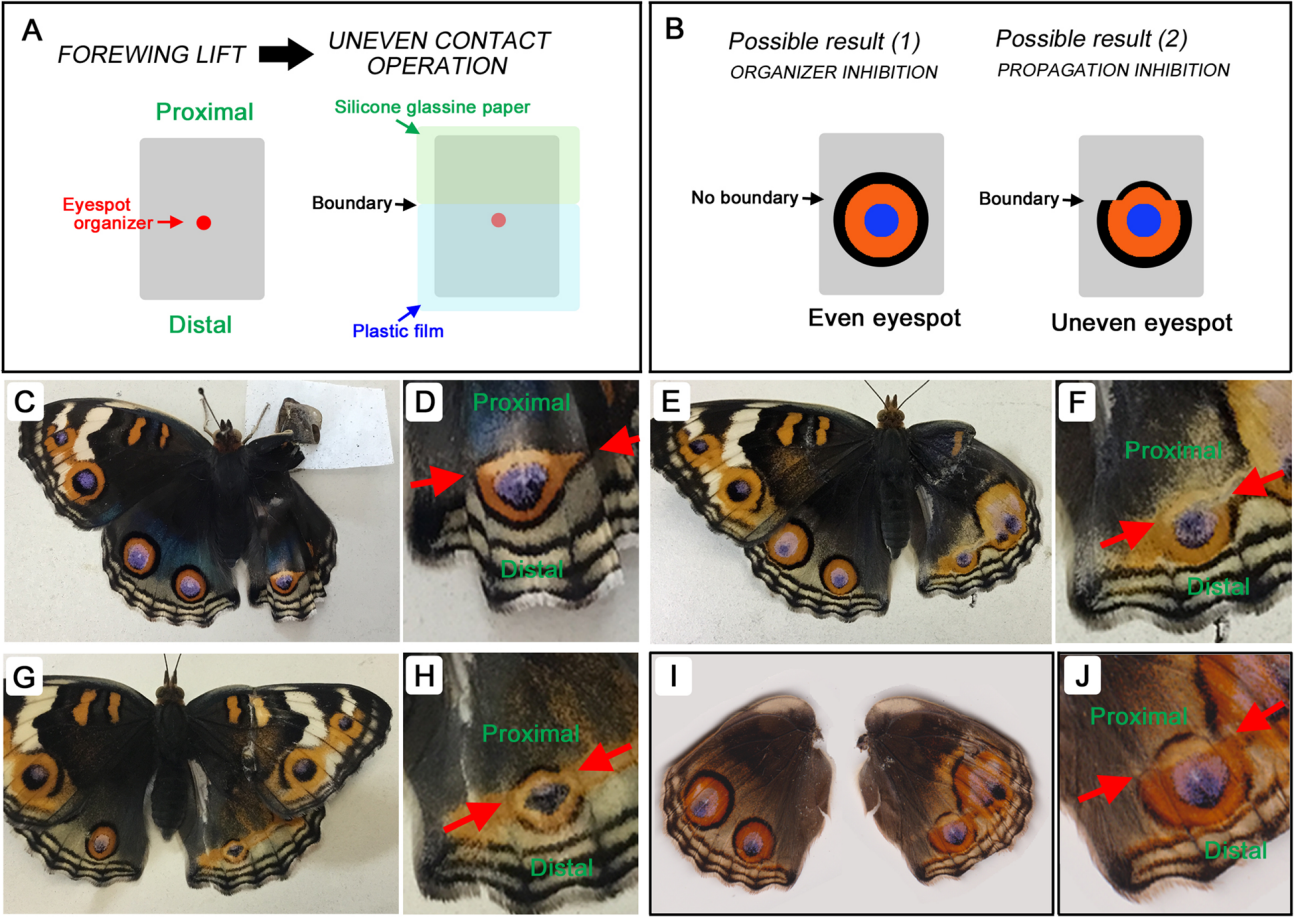
Eyespot responses to two contact materials. **a** Experimental procedure. After the forewing lift procedure, the proximal side of the prospective eyespot was covered with silicone glassine paper, which inhibits eyespot development. The distal side, including the organizer, was covered with plastic film, which does not inhibit eyespot development in most cases. **b** Possible results. If silicone glassine paper inhibits organizing activity, an even eyespot will emerge (left). In contrast, if silicone glassine paper inhibits signal propagation, an uneven eyespot will emerge (right). **c-j** Experimental results. The boundary lines between the two materials are indicated by two opposing red arrows. A treated individual or whole hindwing is shown first (**c**, **e**, **g**, and **i**), and the treated posterior eyespot is enlarged in the following panels (**d**, **f**, **h**, and **j**). The proximal side of the treated eyespot is highly compromised in all cases, but the distal side develops almost normally in **c-f**. In **g** and **h**, the distal side of the treated eyespot is also compromised, although less so than the proximal side. In **i** and **j**, the distal side of the treated eyespot expands, and the proximal side is compromised

Contact materials influence eyespot development by inhibiting either organizer activity to release morphogenic signals or the propagation of morphogenic signals. To distinguish between these two possibilities, an uneven (dual) contact operation was performed (Fig. 9). In one of the contact experiments described above, after a forewing lift, an eyespot organizer and its surroundings were covered with plastic film, which did not greatly affect eyespot size, although enlargements and reductions were also observed. In one of the contact experiments described above, an eyespot organizer together with its surroundings was covered with silicone glassine paper, which almost always resulted in a very small eyespot. Here, the entire distal side and the close vicinity on the proximal side of an organizer were covered with plastic film. The rest of the proximal side was covered with silicone glassine paper (Fig. 9a).

Two possible results can be predicted (Fig. 9b). When the organizer alone (and not the signal propagation) is inhibited by contact with silicone glassine paper, an eyespot of normal size will be produced because the organizer only contacts plastic film and has no contact with silicone glassine paper (Fig. 9b, left). In contrast, when signal propagation is inhibited by silicone glassine paper, the propagating signal from the organizer will not proceed in the area of silicone glassine paper contact, resulting in an uneven eyespot (Fig. 9b, right). The experimental results showed uneven eyespots (*n* = 20); the posterior side was compromised more than the distal side (Fig. 9c-j), suggesting that silicone glassine paper inhibits signal propagation. Because silicone glassine paper is highly water repellant exhibiting a slippery surface, mechanical support from physical contact may be required for eyespot signal propagation.

## Discussion

I tested a variety of contact materials, including biological (e.g., fibronectin) and nonbiological materials (e.g., plastic film, glass plates, and silicone glassine paper) to examine their response profiles, focusing on the hindwing posterior eyespots of the blue pansy butterfly, *J. orithya*. Each material showed a unique profile; for example, plastic film, glass plates, and silicone glassine paper tended to produce no change, enlargement, or reduction of eyespots, respectively. Contact-induced eyespot changes may be unexpected among many researchers because the apical extracellular milieu does not seem to exhibit any chemical connection with molecular morphogens that could specify eyespot colors. Hence, the confirmation and further reinforcement of the previous contact experiment involving *J. almana* [67] in *J. orithya* in the present study are important steps toward understanding eyespot formation mechanisms. Overall, because contact materials function as ECM for epithelial cells, the present study indicates the importance of ECM for eyespot signal propagation. More precisely, nonspecific interactions between ECM and epithelial cells likely mediate signal propagation via mechanical means.

Semiparabiosis and transient lift resulted in similar profiles, which is attributed to the virtually identical contact materials involved. Transient lift produced enlargement or reduction, differing from the results obtained under no treatment. These modifications are probably induced when the hindwing surface does not make even contact with the forewing surface. Appropriate smooth contact without air spaces between the dorsal hindwing epithelium and the ventral forewing epithelium appears to be important for achieving a normal size of the eyespot. Neither enlargement nor reduction after transient lift was observed in *J. almana*; the treated individuals all showed no change [67]. A simple explanation for this discrepancy between species is that the target posterior eyespot in *J. almana* is relatively small, leading to a lower probability of causing uneven contact in the prospective eyespot area in *J. almana*.

Different biological materials showed different response profiles; fibronectin and collagen type I were classified into the “no change group”, gelatin into the “reduction group”, and polylysine into the “enlargement group”, based on scores (Fig. 6). There may be a possibility that fibronectin and collagen type I may be functional, if present, in the prepupal ECM. However, based on change frequencies, plastic plates (i.e., a nonbiological material) produced the highest frequency of no change, indicating that the interactions of epithelial cells with ECM is unlikely specific to particular biological materials.

Among the tested biological materials, polylysine resulted in the highest frequency of enlargement, and its profile was similar to that of glass plates, despite their very different chemical compositions. The glass plates induced the highest frequency of enlargement among the contact materials and treatments examined in the present study. Because glass pipettes have been shown to form a gigaseal with the cell membrane in electrophysiological studies [68, 69] and polylysine is often used in cell culture plates as a cellular binding matrix because of its electrostatic interaction with the cell membrane [70, 71], these two materials may be able to bind tightly to epithelial sheets. This tight binding may cause enlargement of eyespots.

Interestingly, gelatin and water-saturated gelatin showed somewhat similar profiles, but the proportion of enlargement and change ratio were lower in water-saturated gelatin. The addition of water to the gelatin plates probably reduced binding to gelatin because of the disruption of hydrogen bonds and other electrostatic interactions between gelatin and the epithelium by water molecules.

Aluminum foil and alumina file did not show similar results, despite their similar metallic components. This difference may have occurred because they present very different surface structures. Aluminum foil may be able to bind to a cellular sheet because of its even surface, but alumina file does not bind well to an epithelial sheet because of its uneven surface. On the other hand, plastic film and plastic plates showed similar results, possibly because these materials exhibit similar surface structures despite their difference in rigidity. This result suggests that the mechanical rigidity of materials does not contribute greatly to eyespot responses. The relatively low mechanical rigidity of plastic film can suffice as ECM for epithelial cells.

Plastic plates resulted in the highest frequency of no change among the tested contact materials. Semiparabiosis and transient lift led to a lower frequency of no change than plastic plates and plastic film. This finding was somewhat unexpected because the semiparabiosis and transient lift experiments involved the use of fresh actual forewings as the contact material. This result probably reflects the operational difficulties in evenly replacing the lifted forewing in the original position without any wrinkles in the transient lift and semiparabiosis operations. Plastic film has been used for real-time observations of wing development under the assumption that it does not cause substantial changes in color patterns [38, 43, 47]. The present experiments showed that plastic film unfortunately sometimes induced enlargement (or, less frequently, reduction). These results may be explained by the unavoidable operational introduction of uneven binding and distortions of an epithelial sheet.

The following 3 treatments induced a 100% frequency of reduction: medical adhesive tape, copy paper, and no contact. In addition, silicone glassine paper induced an almost 100% reduction frequency. The degree of eyespot size reduction under treatment with these 4 modes was sometimes extreme, resulting in a simple dot or very small eyespot. The results of the no contact experiment directly suggest the need for extracellular contact material for either eyespot signal expansion or organizer activity. The results obtained with medical adhesive tape were somewhat perplexing because the tape is designed to bind well to human skin. However, the tape likely does not bind to the epithelial sheet at all because of its high water repellency. The results observed for copy paper are more perplexing, but cellulose fibers in the paper may be either too rough physically or too neutral chemically to bind to epithelial cells. The results obtained for silicone glassine paper were expected because this type of paper is slippery and does not bind tightly to any surface. These materials and treatments are very different chemically, but they are probably similar in that they do not provide any functional binding matrix for an epithelial sheet because of their high water repellency.

Based on their response profiles, the materials were categorized into 3 groups, as illustrated well in the PCA. Importantly, the response profile and change ratio were significantly correlated with the water contact angle of the material surface. This result suggests that the epithelial sheet requires a reasonably hydrophilic binding surface to form normally sized eyespots.

In the M_3_ compartment (where no eyespot exists without treatment), an ectopic eyespot often emerged, which varied in size (Fig. 8). These ectopic eyespot induction does not appear to be very specific to contact materials. It is likely that various covering materials can activate “sleeping organizer” in the M_3_ compartment, indicating importance of ECM in eyespot development.

Silicone glassine paper could inhibit either the activity of the organizer or signal propagation from the organizer. To distinguish between these two possibilities, the proximal side of a prospective eyespot was covered with silicone glassine paper, and the distal side, including the organizer, was covered with plastic film. Only the proximal side of the eyespot did not expand well, suggesting that silicone glassine paper inhibits signal propagation but not signal release from the organizer. These results suggest that physical contact with an appropriate ECM surface is required for morphogenic signals from organizers to propagate normally in butterfly wings.

When an eyespot was reduced in size by contact with one of the tested materials, such as silicone glassine paper or copy paper, the PFE (an elemental band associated with an eyespot) was simultaneously shifted toward the eyespot focus. In compartments in which eyespots are not present, the PFEs were also shifted toward the proximal side. Both eyespots and PFEs belong to the border symmetry system, and PFEs are equivalent to the eyespot black ring [29, 33, 34]. Somewhat surprisingly, these types of eyespot and PFE modifications are similar, if not identical, to temperature-shock-type (TS-type) modifications. TS-type modifications occur due to temperature shock [15] or the injection of chemicals such as tungstate [16], molsin [17], or heparin and other sulfated proteoglycans [23]. The modifications of PFEs should be incorporated in the model of eyespot formation [33–36, 72]. A minor but interesting finding of the present study was that SMBs were also affected in response to eyespot-reducing contact materials, but SMBs did not appear to be dislocated toward the proximal side. This finding confirms that PFEs and SMBs belong to two different systems: the former belongs to the border symmetry system and the latter to the marginal band system [12].

In the no treatment mode, cuticle secretion appeared to occur into the extracellular space between the forewing and hindwing epithelia because a thin cuticular membrane that covered the hindwing surface was observed when a pharate adult in a pupal case was dissected. In contrast, no such cuticular membrane was observed when most contact materials were placed on the hindwing. Contact stimuli from a variety of materials may inhibit cuticle secretion. Nonspecific contact stimuli may be perceived by cells through integrins, for example. Without the extracellular cuticle membrane or substitute materials, signal propagation may not be possible for epithelial cells.

**Fig. 10:**
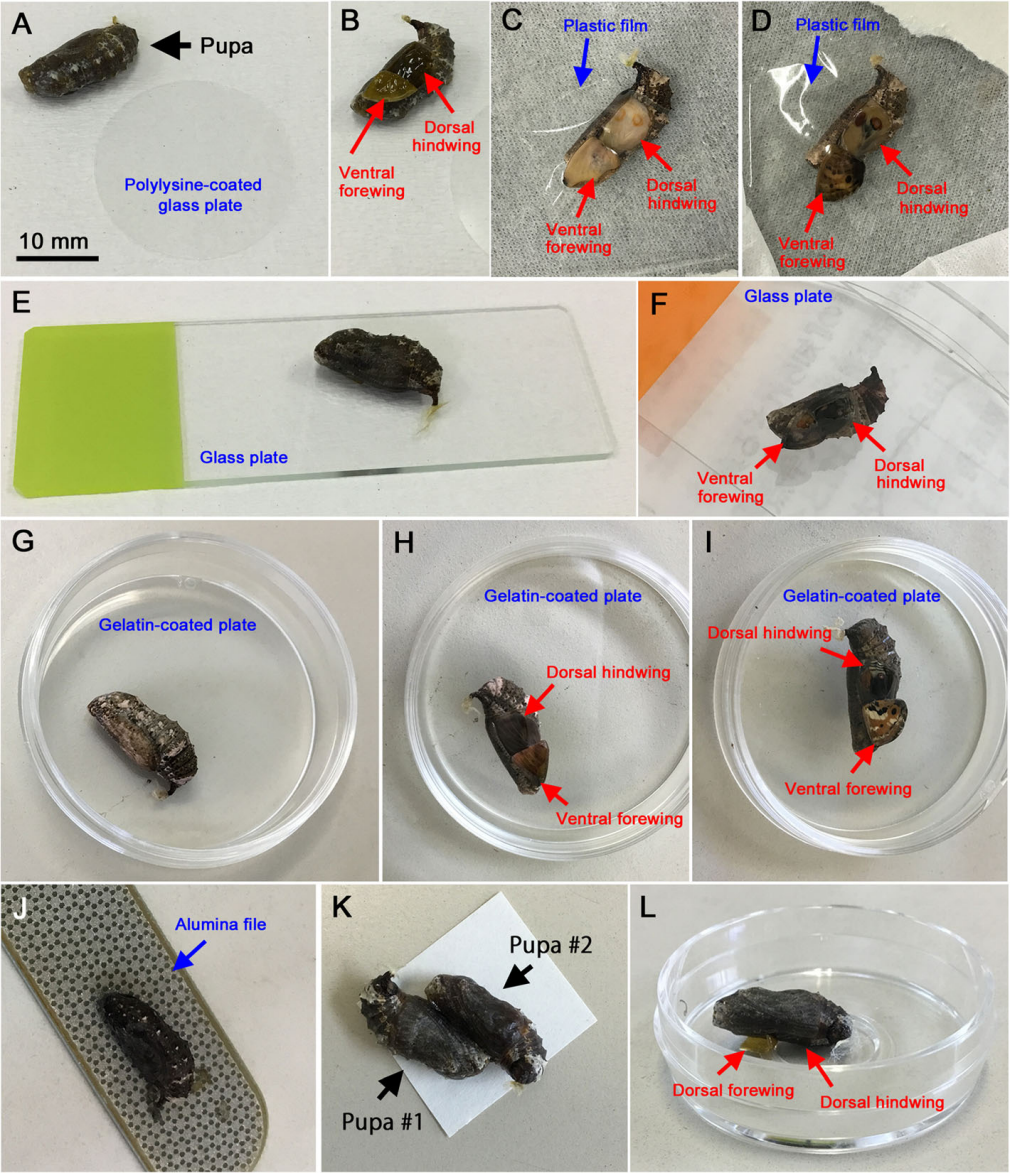
Experimental methods. **a** A freshly pupated pupa and a polylysine-coated glass plate. The scale bar (10 mm) is also applicable to the other panels. **b** A manipulated pupa. The forewing has been lifted, exposing the ventral forewing and the dorsal hindwing. **c** A manipulated pupa covered with a plastic film. The wing color pattern is emerging. **d** Another manipulated pupa covered with a plastic film. The wing color pattern is emerging. The forewing pattern is almost complete, but the hindwing pattern is not. **e** A manipulated pupa on a glass plate. **f** Another manipulated pupa on a glass plate. The ventral forewing and the dorsal hindwing are seen through the glass. Parts of the hindwing that did not contact the glass plate developed a thin cuticle layer. **g** A manipulated pupa on a gelatin-coated plate. **h** A manipulated pupa on a gelatin-coated plate. The ventral forewing and the dorsal hindwing are seen through the glass. **i** Another manipulated pupa on a gelatin-coated plate. The color pattern is almost complete. Parts of the hindwing that did not contact the gelatin-coated plate developed a thin cuticle layer. **j** A manipulated pupa on alumina file. **k** Semiparabiosis. Two pupae with lifted forewings are bound together. **l** A manipulated pupa in a no-contact configuration. Because of the height gap at the center of a glass-bottom dish, the dorsal hindwing made no physical contact

Exceptions to the inhibition of cuticle formation were observed for size-reducing materials (e.g., silicone glassine paper) and no contact treatment. In the no contact treatment, the entire hindwing surface was covered with a thin cuticle membrane (see Fig. 10f, i). Without the formation of this membrane, pupae would die soon due to water loss. Size-reducing materials (i.e., silicone glassine paper, copy paper, medical adhesive tape, and alumina file) also appeared to induce cuticle formation on the surface of the hindwing. Before the formation of the cuticular membrane, morphogenic signals may not be able to propagate. It may be that before the formation of this membrane, signals cannot propagate. In this case, the time-out mechanism of signal settlement [34–36] may function to produce extensively reduced eyespots. That is, if a great deal of time is spent on cuticle formation in the wings treated with these size-reducing materials or no contact, there will be little time left for signals to propagate, resulting in miniaturized eyespots.

Further discussion along these lines could lead to the speculation that eyespot size in natural populations may be genetically regulated at least in part by changing the quality of ECM. A single case of alumina file treatment involved an individual genetic mutant lacking eyespots only on the posterior side (Fig. 4l). This mutant was obtained unintentionally, and all other individuals used in the present study, including those in the semiparabiosis experiment, had discrete posterior eyespots. The use of such an eyespot-less genetic mutant, if established as a line, may be fruitful in the future.

An important question is why nonspecific extracellular mechanical support is required for signal propagation. ECM molecules likely provide mechanical support for the binding of epithelial sheets. The uneven contact experiments demonstrated that contact materials help signals either to propagate or not to propagate normally (Fig. 9). The conventional idea of chemical morphogen propagation does not seem to have any room to incorporate the importance of contact materials (i.e., ECM molecules) in signal propagation. Nonetheless, interactions of epithelial cells with ECM may confer the cells to propagate morphogenic signals. A possible explanation may be given by the distortion hypothesis. The distortion hypothesis states that the primary morphogens that are released from the organizer are physical distortion waves (not chemicals) that travel relatively long distances from the organizer. That is, ECM may function as a supporting material for epithelial distortion. This physical distortion signal may then be translated into chemical signals (i.e., calcium waves) through stretch-activated ion channels or something similar such as Piezo [73]. The calcium waves then trigger molecular morphogens to form a chemical gradient and then execute pigment synthesis. This hypothetical distortion signal propagation is a part of the induction model [36].

Another important question is why the mechanical contact treatments produced color pattern modifications similar to those of temperature-shock and chemical treatments. The targets of tungstate involved in color pattern modifications in butterflies are enigmatic because tungstate is an inhibitor of a large number of enzymes containing molybdopterin (a metal-binding pterin) [74]. Because chemicals probably act extracellularly from inside a wing, there may be a need for mechanical support for epithelial cells on the hemolymph side in wings. Tungstate may inhibit the formation of disulfide bonds that link ECM molecules that cover the basal side of epithelial cells. Temperature shock may also inhibit cuticle formation or disulfide bond formation because this is an enzymatic (temperature-sensitive) step.

In butterfly wings, molecular (chemical) morphogens do not travel along the apical extracellular side, where only cuticle membranes are present, with little liquid medium. Molecular morphogens may therefore travel along the basal (not apical) extracellular side or gap junctions that directly connect cells. However, beyond the ECM layer of the basal side, the hemolymph current may disrupt any gradient of diffusive signals. Cytonemes, originally found in the fruit fly wing imaginal discs [75–77], have been detected on the basal side (but not the apical side) of the pupal wing epithelial cells in butterflies [45, 46] and may play an important role in delivering signaling molecules to distant cells, resulting in a stable gradient of molecular morphogens. Nonmolecular (physical) morphogens, potentially in the form of a mechanical distortion signal of epithelial sheets, may require extracellular support materials to travel. That is, the epithelial sheet is probably sandwiched between the apical extracellular cuticle and the basal ECM molecules, which include many disulfide bonds (e.g., proteoglycans).

In tissue morphogenesis, mechanical forces responsible for cellular shape changes play an important role through tissue bending and invagination [78–83]. The cell-ECM adhesion structures referred to as focal adhesions can transmit force [84], but the ECM to which adhesion structures are bound requires physical rigidity of the ECM for adhesions to mature [85]. Moreover, ECM rigidity is proportional to the forces mediated by focal adhesions [86]. Recently, the propagation of a mechanical morphogenetic wave in *Drosophila* embryogenesis has been demonstrated; waves propagate with positive feedback through local invagination, cell displacement, apical adhesion, contraction, and further invagination [83]. Integrin-mediated adhesion appears to be crucial for wave propagation [83].

## Conclusions

It was demonstrated that eyespot size is influenced by the surface physical chemistry of contact materials. A plausible interpretation of the present experimental results is that morphogenic signals require a cuticle membrane (or substitute materials) to provide mechanical support from the apical side (and from the basal side) of epithelial cells for propagation. The present results are in accordance with the distortion hypothesis, under which epithelial physical distortion serves as a nonmolecular (physical) morphogen.

## Materials and methods

### Butterflies

The blue pansy butterfly, *J. orithya*, was field collected on Okinawa-jima Island or Ishigaki-jima Island, Japan. This butterfly is commonly found on the Ryukyu Archipelago and is not protected. No permission is necessary to catch it in the field or to use it in biological experiments in Japan.

Eggs were collected from field-caught females in the laboratory, and hatched larvae were reared with their natural host plant leaves at ambient temperatures (approximately 27 °C). Alternatively, larvae were field collected. Upon eclosion, adult butterflies were sexed, and only females were subjected to analyses because of the sexual dimorphism of this species. The female forewing shows a simple color pattern including two large eyespots and three peripheral elements (PFEs, SMBs, and marginal bands). No other elements exist. The nomenclature of the elements and subelements described in this paper is shown in Fig. 1. Only the posterior eyespot and its peripheral response were examined in this study. Anterior eyespots were not examined.

### Surgical operations and contact materials

Immediately after pupation, the right forewing was lifted as in previous studies, resulting in the exposure of the ventral forewing and dorsal hindwing surfaces (Fig. 10a, b). Then, contact materials were placed on the surfaces of these wings. Alternatively, the wing surfaces were placed on a material surface. The following contact materials were employed: polystyrene (PS) plates (plastic petri dishes; AS ONE, Osaka, Japan), plastic film (polyvinylidene chloride (PVDC); Kurewrap, Kureha, Tokyo, Japan) (Fig. 10c, d), glass plates (frosted glass slides; AS ONE) (Fig. 10e, f), fibronectin-coated polystyrene plates (60 mm; IWAKI, AGC TECHNO GLASS, Shizuoka, Japan), aluminum foil (DIAMOND, Reynold Consumer Products, Lake Forest, IL, USA), polylysine-coated glass (25 mm; IWAKI) (Fig. 10a), collagen type I-coated glass (25 mm; IWAKI), gelatin-coated polystyrene plates (35 mm; IWAKI) (Fig. 10g-i), alumina-epoxy nail file (100-μm-scale alumina powder embedded with epoxy resin; DAISO INDUSTRIES, Hiroshima, Japan) (Fig. 10j), silicone glassine paper (cooking sheets, CGC Japan, Tokyo), medical acrylic adhesive tape (white tape W129, Nichiban, Tokyo), and copy paper (NEW Yamayuri 100, 70% whiteness, 100% recycled used paper, Oji Paper, Tokyo). The chemical composition of alumina powder is similar to that of aluminum foil, but the surface smoothness or coarseness is different between these two materials. To prevent water loss via water absorbance and evaporation, the manipulated pupae were placed in a small dish (35 mm in diameter) with a lid for a few days when using copy paper as the contact material. Also, wet tissue was placed in the dish.

To examine the possible contribution of water, a gelatin-coated polystyrene dish (35 mm in diameter and 10 mm in height) was saturated with 200 μL deionized water, which was referred to as a water-saturated gelatin plate. For semiparabiosis, two pupae that pupated at the same time were both manipulated together, and the forewing of one individual was placed on the hindwing of another individual (Fig. 10k). In addition to the no treatment control group, a lift control (no contact) was performed. For the lift control, the manipulated pupae were placed in a small dish (35 mm in diameter and 10 mm in height) with a lid to reduce water evaporation. This lid was removed a few days later when the cuticle had completely solidified. The lifted forewing was placed so that it was facing upward or was stuck to the polystyrene portion of a glass-bottom dish (35 mm diameter of the entire dish and 12 mm diameter of the glass portion; IWAKI). In these configurations, the hindwing did not experience any contact (Fig. 10l). For the uneven (dual) contact experiment, the forewing was lifted, and silicone glassine paper and plastic film were placed on the proximal and dorsal sides of a prospective posterior eyespot, respectively.

The manipulated pupae were placed in a plastic container (78 mm diameter at the bottom and 55 mm height; Chuo Kagaku, Saitama, Japan) at ambient temperature (approximately 27 °C) until eclosion. Representative adult butterflies were photographed when they were alive except in the no control and plastic film experiments. Thereafter, they were frozen in a freezer to prevent the wings from being damaged.

### Evaluation of color pattern changes

Responses to a contact material were evaluated on the basis of the size of the manipulated (right) posterior dorsal hindwing eyespot in comparison to the nonmanipulated (left) eyespot. The percentages of the 3 categories were calculated for each treatment and were referred to as response profiles. Simultaneously, the eyespot was judged to present an increase (+ 1.00), a decrease (− 1.00), or no change (0.00) according to visual inspection, and scores were accordingly assigned for these categories. The final score for each material was calculated as the average of these individual scores together with the standard error values as follows: glass plate (0.38 ± 0.16; *n* = 21), plastic film (0.29 ± 0.08; *n* = 38), transient lift (0.31 ± 0.09; *n* = 42), semiparabiosis (0.25 ± 0.11; *n* = 16), polylysine (0.19 ± 0.20; *n* = 21), aluminum foil (0.11 ± 0.10; *n* = 53), plastic plate (0.13 ± 0.10; *n* = 23), collagen type I (0.04 ± 0.17; *n* = 27), fibronectin (0.00 ± 0.14; *n* = 26), no treatment (0.00 ± 0.00; *n* = 19), gelatin (− 0.22 ± 0.11; *n* = 46), alumina file (− 0.41 ± 0.11; *n* = 46), water-saturated gelatin (− 0.58 ± 0.10; *n* = 31), silicone glassine paper (− 0.96 ± 0.04; *n* = 26), copy paper (− 1.00 ± 0.00; *n* = 42), medical adhesive tape (− 1.00 ± 0.00; *n* = 20), and no contact (− 1.00 ± 0.00; *n* = 20). The changes in the anterior eyespot were observed when apparent but were not scored, even if it changed dramatically because the anterior eyespot cannot be covered completely with the contact materials due to its curvature and unevenness at the pupal surface, as shown in a previous study [67]. Other overall color pattern changes were recorded qualitatively.

To measure eyespot size, the right and left hindwings were separated from the body. When necessary, a piece of glass slide was placed onto the wing so that it was flattened. The posterior hindwing size was measured in the proximodistal direction on the midline (being parallel with the wing veins) under a desktop digital microscope SKM-S30A-PC and its associated software SK measure (Saitoh Kougaku, Yokohama, Japan). Eyespot size change ratio was expressed by the treated eyespot size divided by the corresponding nontreated (control) eyespot size as follows (mean ± standard deviation): glass plate (1.148 ± 0.218; *n* = 21), polylysine (1.093 ± 0.106; *n* = 21), aluminum foil (1.054 ± 0.275; *n* = 50), plastic plate (1.050 ± 0.114; *n* = 24), plastic film (1.042 ± 0.050; *n* = 38), collagen type I (1.042 ± 0.252; *n* = 27), transient lift (1.034 ± 0.103; *n* = 40), semiparabiosis (1.013 ± 0.108; *n* = 16), no treatment (0.999 ± 0.025; *n* = 19), fibronectin (0.999 ± 0.154; *n* = 25), gelatin (0.993 ± 0.144; *n* = 23), alumina file (0.841 ± 0.228; *n* = 30), water-saturated gelatin (0.837 ± 0.118; *n* = 14), no contact (0.739 ± 0.135; *n* = 18), medical adhesive tape (0.696 ± 0.146; *n* = 18), silicone glassine paper (0.678 ± 0.132; *n* = 25), and copy paper (0.641 ± 0.194; *n* = 36). The change ratio were then subjected to statistical analysis.

### Contact angle measurements

Water contact angles were measured using an ASUMIGIKEN ME2 contact angle meter (Tokyo, Japan). This contact angle meter employs a half-angle method and calculates the contact angle in real time from a droplet image. Its resolution is 0.01°. The contact angle is a function of the wettability (or repellency) of a material surface and it is directly related to adhesiveness to hydrated materials. A 25-gauge needle filled with deionized water was placed in the contact angle meter, and a single 1.0-μL droplet was slowly placed on a material surface while being monitored on a computer screen. Droplet shapes were visually checked. Immediately after the placement of the droplet (within 1 min), measurements were quickly performed 10 times in a row. Quick measurement was critical because of continuous water evaporation and droplet shape changes over time. The mean value for these 10 measurements was considered to represent a single trial (*n* = 1). For each material, 10 trials were performed (*n* = 10), the values were averaged, and the standard deviation was calculated. The following results were obtained: adhesive tape (119.3 ± 5.7°), silicone glassine paper (112.8 ± 1.0°), copy paper (110.6 ± 6.1°), alumina file (109.0 ± 6.2°), aluminum foil (108.5 ± 8.7°), plastic plate (95.3 ± 6.2°), semiparabiosis (91.1 ± 8.5°), fibronectin (86.1 ± 7.1°), gelatin (69.4 ± 1.6°), polylysine (67.6 ± 1.5°), collagen type I (65.1 ± 3.1°), plastic film (64.8 ± 4.3°), and glass plates (23.2 ± 6.6°).

### Statistical analyses

Correlation analysis, PCA, and *t*-test (unpaired, bi-sided) were performed using JSTAT 13.0 (Yokohama, Japan). Either Student’s or Welch’s *t*-test was performed, according to *F*-test. Results of *t*-test were not adjusted in multiple testing between control (no treatment) and experimental groups (Fig. 6e). But for Bonferroni method, correction factor was 16. Scatter plots and other graphs were drawn using Microsoft Excel 2013. For principal component analyses, the percentages of the 3 categories (enlargement, reduction, and no change) were used as input variables.

## Supplementary information

**Additional file 1: Table S1.** Eyespot change scores for treatments. **Table S2.** Eyespot change percentages and contact angles for materials. **Table S3.** Eyespot size change ratios for treatments.

## Data Availability

All data generated or analyzed during this study are included in this published article and its supplementary information file.

## Abbreviations

CRISPR/Cas9: Clustered regularly interspaced short palindromic repeats / CRISPR-associated protein 9
ECM: Extracellular matrix
*J.*: *Junonia*
PFE: Parafocal element
PCA: Principal component analysis
RNAi: RNA interference
SMB: Submarginal band
TS-type: Temperature shock-type
